# Case of an abnormal procalcitonin‐producing metastatic pancreatic neuroendocrine tumor

**DOI:** 10.1002/ccr3.3187

**Published:** 2020-09-17

**Authors:** Wei‐Zhen Wang, Long‐Jiang Xu, Duan‐Min Hu, Wen Tang

**Affiliations:** ^1^ Department of Gastroenterology The Second Affiliated Hospital of Soochow University Suzhou China; ^2^ Department of Pathology The Second Affiliated Hospital of Soochow University Suzhou China

**Keywords:** biomarker, liver metastatic, pancreatic neuroendocrine tumor, procalcitonin

## Abstract

Procalcitonin (PCT) is widely used to diagnose a bacterial infection. An increased serum PCT can also be observed in tumors. We presented an unusual case of a metastatic PNET producing and secreting PCT. Immunohistochemistry was used to demonstrate that PCT can be secreted by PanNET.

## Graphical abstract

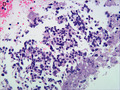

To the Editor,

Pancreatic neuroendocrine tumors (PanNETs) are a rare heterogeneous group of malignancies caused by amine precursor uptake and decarboxylation (APUD) cells that secrete various hormones. Chromogranin A (CgA) is considered the most useful neuroendocrine tumor biomarker to help diagnose this disease and follow‐up its evolution. Unfortunately, many hospitals cannot measure serum CgA in China. Hence, new biomarkers should be developed to detect tumors early, evaluate patients' medical treatment response to APUD, and monitor disease progression. Herein, we presented an unusual case of a metastatic PanNET producing and secreting procalcitonin (PCT), which may become a useful circulating biomarker.

## 1 CASE REPORT

In February 2017, a 64‐year‐old woman suffered from fatigue, decreased appetite, diaphoresis with cool skin, pale appearance, and palpitation, resulting in spontaneous remission after 10 minutes. The attacks were irregular. The patient was admitted to other hospitals presenting these symptoms. Abdominal ultrasound and contrast‐enhanced computed tomography revealed multiple liver lesions and a mass between the pancreas and the spleen (imaging unavailable). The patient was not treated with a specific therapy because of the absence of pathological findings. In September 2017, the patient suffered from diarrhea for approximately 7‐10 times/day and had watery stool. On October 4, 2017, the patient presented to our hospital with a weight loss of 12.5 kg in the past 8 months. Her detailed physical examination results were unremarkable. She was taking metoprolol to treat hypertension that she had for 20 years. She also had diabetes for 8 years and was treated with insulin.

**FIGURE 1:**
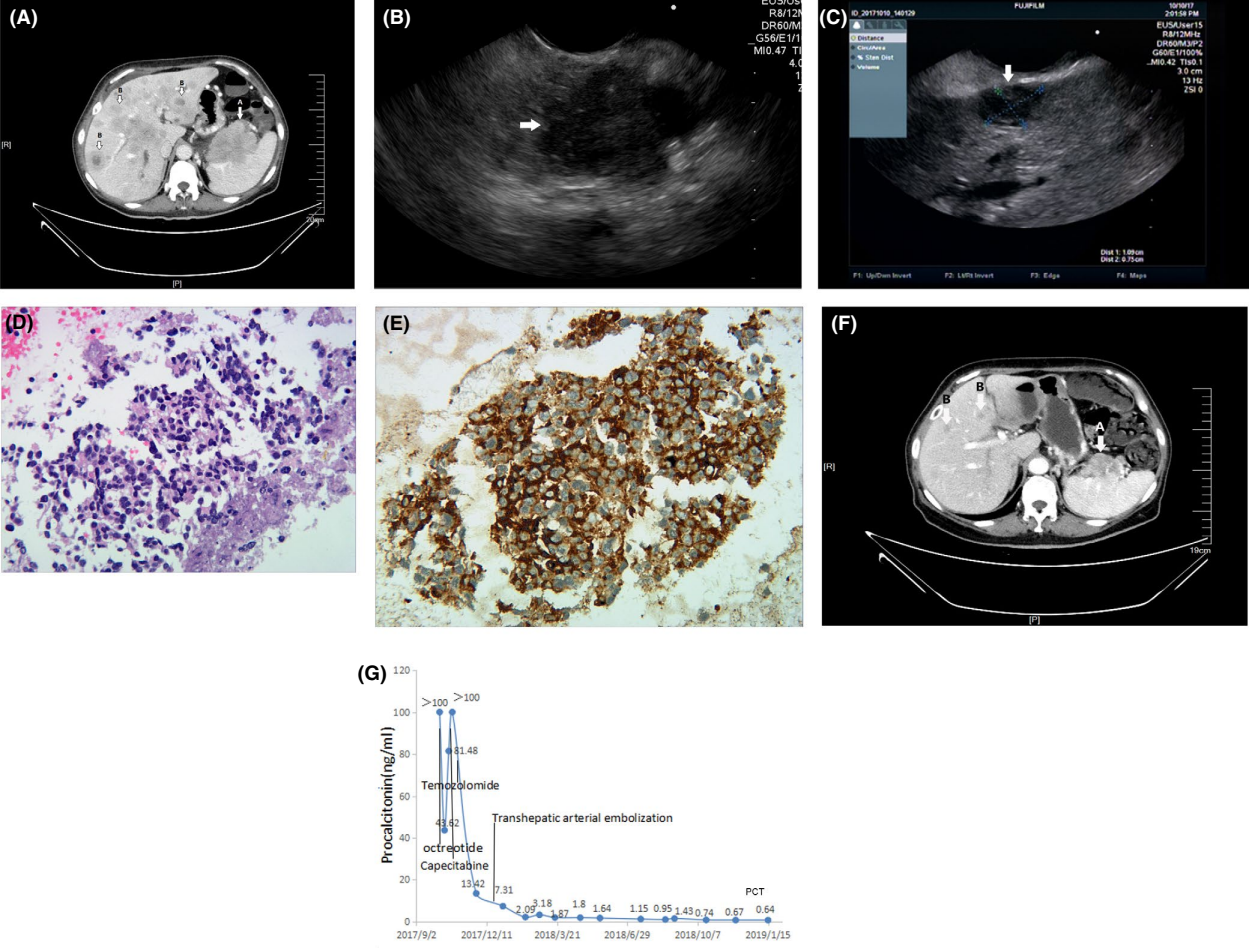
A, Contrast‐enhanced computed tomography before treatment demonstrated a mass in front of the hilum of the spleen (A) and multiple liver lesions (B). B and C, Endoscopic ultrasound revealed a pancreatic mass (B) and multiple liver lesions (C). D, Routine hematoxylin‐eosin staining of a pancreatic specimen and multiple liver lesions revealed small round cell tumors that stained blue (Original magnifications, ×400). E, Immunohistochemical staining for procalcitonin monoclonal antibody was positive (Original magnifications, ×400). F, After the patient received the initial treatment, serum PCT level considerably decreased from >100 ng/mL to 2.09 ng/mL and remained low. G, Contrast‐enhanced computed tomography after 4 mo of treatment demonstrated a decrease in pancreatic mass (A) and multiple liver lesions (B)

Contrast‐enhanced computed tomography of the chest, abdomen, and pelvis demonstrated an 88 mm × 60 mm mass in front of the hilum of the spleen and multiple liver lesions (Figure 1A). Endoscopic ultrasonography demonstrated a large mass at the tail of the pancreas and multiple liver masses (Figure 1B,C). The patient then underwent an endoscopic ultrasound‐guided fine‐needle aspiration of the liver lesion and the tail of the pancreatic lesion. Pathological results revealed small round cell tumors (Figure 1D). Further immunohistochemical staining of the pancreatic and hepatic specimens showed Ki‐67 stain in 5% positive cells, chromogranin A (CgA)‐positive, synaptophysin‐positive, and CK56‐positive. The stains for CK7, CK20, CK19, S100, villin, hepatocyte, AFP, and P53 were negative. Consequently, the histologic diagnosis of PanNET (grade 2, World Health Organization [WHO], 2017)^1^ was confirmed. Bone scintigraphy with technetium 99 was performed, and no abnormalities were found.

Laboratory tests, such as blood routine and hepatorenal function, were within their normal range except a decreased hemoglobin of 91 g/L (normal, 115‐150 g/L). Tumor markers were negative for carcinoembryonic antigen, alpha‐fetoprotein, carbohydrate antigen 19‐9, and neuron‐specific enolase but were positive for carbohydrate antigen 125 (80.31 U/m [normal, <35]) and calcitonin (>2000 pg/mL [normal, <5.0]). Insulin, adrenocorticotrophic hormone, cortisol, prolactin, and other endocrine hormones, thyroid function, and serum calcium were normal. Unfortunately, CgA, serotonin (or 5‐hydroxytryptamine), and urine 5‐hydroxyindoleacetic acid measurements were unavailable in our hospital. The patient never experienced hypoglycemic events, peptic ulcer, and osteoporosis. Hence, multiple endocrine neoplasia was excluded.

Laboratory examination showed an abnormally increased serum procalcitonin (>100 ng/mL [normal, 0‐13.8]). However, the patient did not experience other clinical manifestations, such as fever, cough, expectoration, abdominal pain, and cervical lymphadenectasis. Leukocytes, neutrophils, and CRP were also normal. To investigate whether tumor cells were the origin of the abnormally increased serum PCT, we reviewed the pancreatic and hepatic biopsy specimen. Immunohistochemical staining for procalcitonin monoclonal antibody (44d9, Novus Biologicals, USA) showed that PCT was expressed and secreted by the tumor cells (Figure 1E).

In the end, we treated the patient diagnosed with PanNETs with liver metastases (G2, WHO Classification 2017).^1^ Octreotide acetate (200 μg sc bid) was used to control her symptoms. After receiving octreotide acetate, the patient was asymptomatic. Given that the patient with the metastatic disease was symptomatic, she was treated with capecitabine (1000 mg, po, bid) on days 1 to 14 and temozolomide (200 mg, po, qd) on days 10 to 14 every 28 days. The patient underwent hepatic transcatheter arterial embolization thrice for hepatic‐predominant disease during systemic chemotherapy. All of the treatments were well tolerated except mild fatigue. To investigate the association between serum PCT level and treatment response, we followed up serum PCT regularly. The evolution of the serum PCT level in this case is presented in Figure 1F. After 4 months of the initial treatment, the repeated contrast‐enhanced computed tomography of the patient showed a decrease in pancreatic mass and multiple liver lesions (Figure 1G). The patient achieved stable disease (SD) in accordance with RECIST criteria. At present, the patient is still being treated in our hospital.

## 2 DISCUSSION

Pancreatic neuroendocrine tumors can be classified as functional and nonfunctional based on their ability to secrete hormones and elicit distinct symptoms. The majority of patients with PanNET (90.8%) are nonfunctional pancreatic neuroendocrine tumors (NF‐PETs).^2^ Most NF‐PETs can secrete various peptides, such as pancreatic polypeptide, neuron‐specific enolase, CgA, and human chorionic gonadotropin subunits, which do not manifest with distinct clinical symptoms. Therefore, the diagnosis of NF‐PETs may be delayed for many years with large primaries (70% > 5 cm) and liver metastases (>60%).^3^ Hence, circulating neuroendocrine tumor biomarkers can help detect tumors early. CgA is currently the best serum biomarker to help diagnose diseases, evaluate therapeutic response, and predict prognosis in NET. Despite its usefulness, the use of CgA is limited because many hospitals in China cannot measure serum CgA. Therefore, finding new biomarkers is promising.

In this patient with PanNET (G2, WHO classification 2017),^1^ we observed an abnormally high level of serum PCT (>100 ng/mL [normal, 0‐0.05]) without a bacterial infection. PCT, a 116‐amino acid polypeptide encoded by the CALC gene, is widely used to diagnose a bacterial infection. An increased serum PCT can also be observed in tumors, such as MTC, in patients with primary lung cancer and a neuroendocrine component, and in any tumor with liver metastases. Hence, we further studied the correlation between increased serum PCT and tumor cells. The immunohistochemical staining of procalcitonin revealed that PCT was secreted by the tumor cells. A previous case also reported PCT‐secreting PanNET cells, and this finding is confirmed through immunohistochemical staining.^4^ However, Chen L et al revealed that the immunohistochemical staining of PCT was positive only in 24% (23 of 96 patients with NET of the digestive system).^5^ Future studies should determine whether increased serum PCT is secreted by PanNET cells.

Our case was unique because we followed up serum PCT to investigate the correlation of serum PCT level and treatment response. We observed that the serum PCT level considerably decreased from >100 ng/mL to 2.09 ng/mL after the initial treatment was administered. The patient achieved SD after 4 months. During follow‐up, we found that pancreatic mass and multiple liver lesions were gradually reduced, and low PCT levels were retained. We would continue the follow‐up to determine whether the serum PCT level would change when patients experienced disease progression. Chen L et al revealed that serum PCT increases in the NET of the digestive system, especially in patients with a high tumor burden; this finding could help evaluate treatment response, provide disease prognosis, and monitor tumor progression.^5^ Procalcitonin may become a promising tumor biomarker for gastrointestinal PanNET. Further multicenter studies with a large sample are needed to verify the correlation of serum PCT level and tumor burden, treatment response, and tumor progression.

In summary, we presented the first case that strictly followed up the evolution of the serum PCT level and size of lesions in a patient with PCT‐secreting metastatic PanNET (G2). Immunohistochemistry was used to demonstrate that high level of PCT can be secreted by PanNET. After receiving treatment, the patient showed a decrease in pancreatic mass and multiple liver lesions and a remarkable decrease in serum PCT level. However, further studies are needed to ascertain the potential link between PCT changes and clinical course in patients with PanNET.

## CONFLICT OF INTEREST

There are no conflicts of interest. The manuscript has been read and approved by all the authors.

## AUTHOR CONTRIBUTIONS

WW: involved in study design, literature research, clinical studies, data acquisition, data analysis/interpretation, manuscript preparation, manuscript editing. WT: was guarantor of integrity of entire study, involved in study concepts, manuscript revision/review, manuscript final version approval. DH: was guarantor of integrity of entire study, involved in study concepts, study design, data analysis/interpretation, manuscript definition of intellectual content, manuscript editing. LX: involved in literature research, experimental studies, manuscript preparation, manuscript preparation, manuscript editing.

## CONSENT STATEMENT

The authors certify that they have obtained all appropriate patient consent forms. In the form, the patient has given her consent for her images and other clinical information to be reported in the journal. The patient understand that her name and initials will not be published and due efforts will be made to conceal their identity.

## Data Availability

No additional data available.

## References

[1] Perren A , Couvelard A , Scoazec JY , et al. ENETS consensus guidelines for the standards of care in neuroendocrine tumors: pathology: diagnosis and prognostic stratification. Neuroendocrinology. 2017;105(3):196‐200. 28190015 10.1159/000457956

[2] Halfdanarson TR , Rabe KG , Rubin J , Petersen GM . Pancreatic neuroendocrine tumors (PNETs): incidence, prognosis and recent trend toward improved survival. Ann Oncol. 2008;19(10):1727‐1733. 18515795 10.1093/annonc/mdn351 PMC2735065

[3] Metz DC , Jensen RT . Gastrointestinal neuroendocrine tumors: pancreatic endocrine tumors. Gastroenterology. 2008;135(5):1469‐1492. 18703061 10.1053/j.gastro.2008.05.047 PMC2612755

[4] Hagiya H , Matsui T , Kitamura T , et al. Pancreatic neuroendocrine tumor abnormally secreting procalcitonin. Pancreas. 2017;46(1):E7‐E9. 27977635 10.1097/MPA.0000000000000708

[5] Chen L , Zhang Y , Lin Y , et al. The role of elevated serum procalcitonin in neuroendocrine neoplasms of digestive system. Clin Biochem. 2017;50(18):982‐987. 28668469 10.1016/j.clinbiochem.2017.06.010

